# First evaluation of bendiocarb in experimental huts using different substrates in Madagascar

**DOI:** 10.1186/s12936-016-1345-z

**Published:** 2016-05-26

**Authors:** Sanjiarizaha Randriamaherijaona, Thiery Nepomichene, Jade Assoukpa, Yoann Madec, Sébastien Boyer

**Affiliations:** Unité d’Entomologie Médicale, Institut Pasteur de Madagascar, BP 1274, Antananarivo, Madagascar; Ecole Doctorale Sciences de la Vie et de l’Environnement, Université d’Antananarivo, Antananarivo, Madagascar; Unité d’Epidémiologie des Maladies Emergentes, Institut Pasteur, 25-28, rue du Docteur Roux, 75015 Paris, France

**Keywords:** Bendiocarb, Residual activity, Bioassay, Carbamate, Insecticide, Madagascar, *Anopheles arabiensis*, *Aedes albopictus*, malaria vector

## Abstract

**Background:**

Indoor residual spraying with insecticide is recommended for malaria control in high-transmission settings. Determination of residual activity of insecticides is essential for the selection of appropriate indoor spraying policy. The present study was undertaken to evaluate the residual effect of bendiocarb, a carbamate insecticide used in Madagascar, on different indoor surfaces in order to elaborate future vector control interventions.

**Methods:**

The residual activity of bendiocarb was evaluated in both experimental huts and houses. Tests in experimental huts on different substrates represented a small scale-field trials. The houses IRS performed in parallel of experimental huts IRS, was done to compare semi-field results and field results. Bioassays according to the World Health Organization (WHO) standard protocol were carried out on different substrates impregnated with bendiocarb using susceptible strains of *Anopheles arabiensis* and *Aedes albopictus*.

**Results:**

Bendiocarb induced significantly high mortality in treated huts against exposed mosquito (p < 0.005) compared to untreated huts. The mortality is up to the WHO threshold of 80 % during 5 months post-treatment. Using a multivariate analysis, *Ae. albopictus* mortality decreased significantly from the 3rd month post-treatment. However, *An. arabiensis* mortality decreased significantly from the 4th month after treatment. Comparing mosquito mortality results from the mud experimental huts and the mud houses showed no significant difference regarding the persistence of bendiocarb on wall.

**Conclusions:**

Current data suggest variable persistence of bendiocarb according to the type of wall surfaces, highlighting the importance of testing insecticide for IRS in local context before using them in large scale. Data from this study validate also the importance of using experimental huts as representative tool to evaluate the effectiveness of an insecticide.

## Background

An efficient control of vector species is central in malaria eradication policy [1]. Indoor residual spraying (IRS) formed the mainstay of the vector control activities in the southern African sub-region. Initially, IRS was considered useful for malaria prevention in areas with low-to-moderate transmission, whereas insecticide-treated nets (ITNs) were considered as suitable in high endemic areas [2]. Operational scale malaria vector control using IRS and ITNs has been implemented extensively in most malaria endemic countries [3, 4]. Pyrethroids are the most commonly used insecticides for net impregnation due to their efficacy, fast acting effect at low dose and low toxicity for mammals [5].

The implementation of insecticide resistance management strategies is necessary to avoid their development in malaria vectors which may compromise the success of vector control, and to preserve the efficacy of used insecticides [6]. Currently, the IRS efficacy of contemporary insecticides recommended by the World Health Organization (WHO) is highly variable. Although dichlorodiphenyltrichloroethane (DDT) is both long-lasting and cost-effective [7]. However, organophosphates, carbamates, and pyrethroids that are ideal for IRS are mostly shorter-lived and more expensive.

Actually, carbamates are effective insecticides to control malaria vectors in pyrethroid-DDT resistance areas, mainly because of its different mode of action, and no cross- or multiple-resistances are reported until now. In Benin, a decrease of malaria transmission was observed in the months following a large-scale IRS campaign using the carbamate insecticide bendiocarb, protecting more than 350,000 peoples [8, 9]. In the Gambia, mosquito house entry, estimated by light traps, was significantly lower in houses sprayed by bendiocarb than in unsprayed houses [10]. Regarding this proved efficacy of bendiocarb, the Malagasy Republic adopted a national malaria control strategy based on large-scale integrated control measures with IRS of bendiocarb since 2009. Previous studies reported that residual life of an insecticide depends on the substrate on which it is applied [11, 12].

In the present study, a monthly following of bendiocarb residual activity was conducted to investigate its residual life on different substrates. This study performed with sensitive *Anopheles* and *Aedes* strains was carried out under small scale-field conditions in experimental huts and in field conditions in inhabited houses to compare the semi-field and field results.

## Methods

### Biological materials for insecticide residual activity monitoring

Two laboratory strains were used for all insecticide tests: *Anopheles arabiensis* and *Aedes albopictus*. Those strains were respectively established in January 2008 and in April 2010 at Institut Pasteur de Madagascar. Those colonies were characterized in the laboratory for insecticide susceptibility using standard WHO impregnated paper tests: 100 % mortality was observed with DDT, fenitrothion, propoxur, permethrin, deltamethrin and bendiocarb. No *kdr* or *ace*-*1* resistance gene was detected by PCR in *An. arabiensis*.

### Study sites and substrates treated

**Fig. 1:**
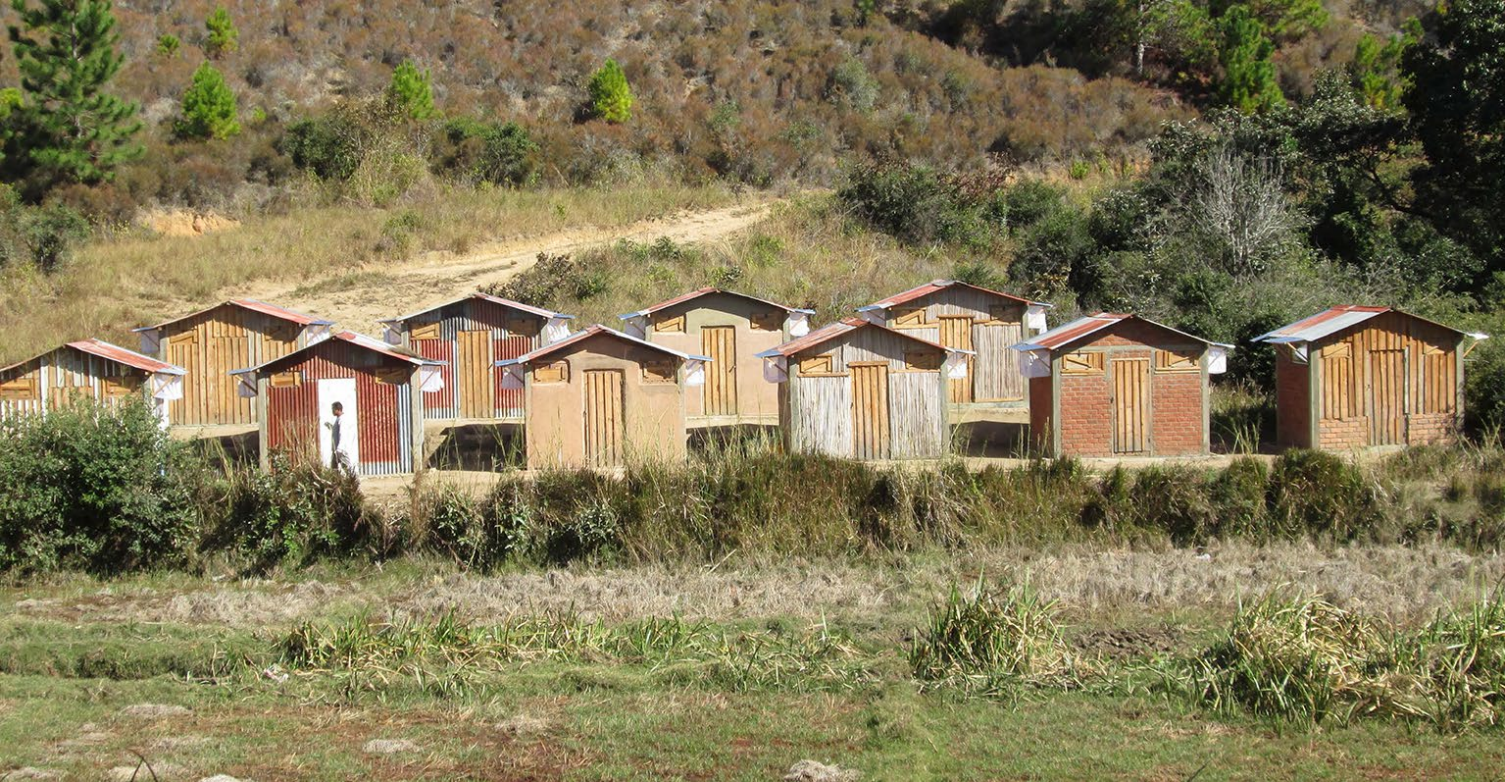
Experimental huts. Saharevo station

Located on the eastern edge of the Malagasy Central highlands, villages of Saharevo (S18°51′12.9″ E48°07′48.8″) and Ambohitranivo (S18°50′52.71″ E48°14′24.17″) are located in the Moramanga district, Madagascar. Ten experimental huts were built with different wall type representing the different habitat types in Madagascar (walls made of cement, wood, tin, mud and vegetal materials). Each hut type was duplicated, one being the control hut and the other the treated hut (Fig. 1).

### Insecticide and insecticide treatment

A wettable powder formulation of bendiocarb (80 WP Ficam ^®^) provided by the Malagasy National Malaria Control Programme (NMCP) was used. This insecticide is an irreversible acetylcholinesterase inhibitor acting on the insect central nervous system [13]. Bendiocarb WP 80 % is among the 12 insecticides recommended by WHO for indoor residual spraying against malaria vectors with residual activity estimated to 6 months [14]. It was used at the WHO recommended dose of 80 % (0.4 g/m^2^). The spraying was performed by qualified NMCP’s agents according to WHO recommendations [15].

The spraying of the experimental huts was performed in one day at the end of July 2013, at the middle of the dry season after 2 months of bioassay with no insecticide, acting as a control period. It also allows that the experimental huts are not contaminated by insecticides [7]. The same day, fifteen randomly chosen houses per village were also sprayed. The insecticide treatment was performed under similar conditions. All experiment measurements were performed (in experimental huts and in houses) from June 2013 to February 2014.

### Residual activity of insecticide treatment

The evaluation of insecticide treatment started 1 month after treatment and ran for 7 months from August 2013 to February 2014. WHO cone bioassay test was undertaken in every hut and house walls to evaluate the residual activity of the insecticide. Every month, 10 3–5 day-old females of *An. arabiensis* or *Ae. albopictus* were introduced into each cone. One cone was fixed to each face of wall per hut for 30 min exposure according to WHO guidelines [7], thus a total replicate of four cones were used per house. Then, mosquitoes were transferred into a small plastic cup for holding. 10 % sugar solution was provided during the 24 h holding period at 25 °C and 80 % relative humidity in the insectarium. After 24 h, alive and dead mosquitoes were recorded.

### Data analysis

The unit of all statistical analyses was the cone. In the experimental huts, two sites, during 9 months, with 10 huts and four cones per hut lead to 720 units of measurement; 440 were observed without treatment, and 280 with treatment. In houses, during 9 months, with 22 houses with four cones in each house lead to 792 units of measurement; 176 without treatment and 616 with treatment. Of the 15 houses from each site, 10 and 12 were in mud and only those were retained in the analysis. All analysis was conducted separately for each mosquito species.

Mortality was defined as the proportion of mosquitoes who had died per statistical unit, and was described using median, inter-quartile range (IQR) and range. Comparisons between treated and untreated huts on the one hand, and treated and untreated houses on the other hand were conducted using the Wilcoxon or Kruskal–Wallis non parametric tests, as distributions were not Gaussian. Overall comparisons, and comparison after controlling for site, type of wall, or month were performed.

Treatment is considered effective if it leads to a mortality of at least 80 %. Each unit was then defined based on his threshold, and a logistic regression model was implemented to evaluate whether the type of wall modified the effect of the treatment, and if the effect of treatment faded with time (tested using interactions). In order to account for repeated measurements on similar units (hut or house), random effects were introduced. Statistical analysis were performed using R [16].

## Results

A total of 3024 bioassays were performed during 9 months of follow-up using 15,120 females of *An. arabiensis* and 15,120 females of *Ae. albopictus*. For each species, 616 measurements were carried out on untreated walls (440 in experimental huts and 176 in houses) and 896 measurements on treated walls (280 in experimental huts and 616 houses).

When restricting the analysis to untreated huts, mortality was similarly low whatever the type of walls both in *An. arabiensis* and *Ae. albopictus*. The mortality rates recorded were always below 5 % (average = 2.6 %).

**Fig. 2:**
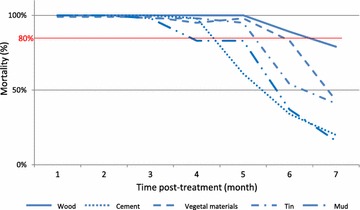
Persistence of bendiocarb on different walls in experimental huts against *An. arabiensis*

**Fig. 3:**
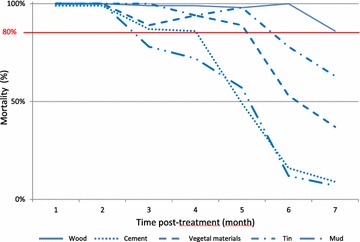
Persistence of bendiocarb on different walls in experimental huts against *Aedes albopictus*

For both mosquito species, in experimental huts, the insecticide treatment on walls increased significantly the mosquito mortality rate (p < 0.0001) regardless of the type of wall and the time to treatment (Tables 1, 2). For *An. arabiensis*, the mortality rate was 98–100 % during 3 months post-treatment on different treated substrates. From the 4th to the 7th month, induced mortality ranged from 16 % (wall made of mud) to 100 % (wall made of wood), indicating a difference in the persistence of insecticide based on treated substrate (Fig. 2). Regarding bioassay with *Ae. albopictus* based on wall type, mortality rate is up to 80 % (WHO threshold validity) during three months when bendiocarb is applied on mud wall. *Aedes albopictus* mortality breaks through the threshold of 80 % after the 4th, the 5th, the 6th and the 7th months post-treatment respectively for cement, vegetal materials, tin and wood wall types (Fig. 3).

**Table 1:** Univariate analysis of induced mortality against *An. arabiensis* in experimental huts

Variables	Untreated huts			Treated huts			*p value*
	N	Median [IQR]	[Min–max]	N	Median [IQR]	[Min–max]	
		[IQR]			[IQR]		
Treatment	440	0.0 [0.0–0.0]	[0.0–1.0]	280	1.0 [0.7–1.0]	[0.0–1.0]	<2.0 10^−16^
Site							
Ambohitranivo	220	0.0 [0.0–0.0]	[0.0–0.3]	140	1.0 [0.8–1.0]	[0.0–1.0]	<2.0 10^−16^
Saharevo	220	0.0 [0.0–0.0]	[0.0–1.0]	140	1.0 [0.7–1.0]	[0.1–1.0]	<2.0 10^−16^
Wall type							
Wood	88	0.0 [0.0–0.0]	[0.0–0.1]	56	1.0 [1.0–1.0]	[0.5–1.0]	<2.0 10^−16^
Cement	88	0.0 [0.0–0.0]	[0.0–0.2]	56	1.0 [0.3–1.0]	[0.0–1.0]	<2.0 10^−16^
Vegetal materials	88	0.0 [0.0–0.0]	[0.0–0.3]	56	1.0 [0.9–1.0]	[0.2–1.0]	<2.0 10^−16^
Tin	88	0.0 [0.0–0.0]	[0.0–0.2]	56	1.0 [0.7–1.0]	[0.1–1.0]	<2.0 10^−16^
Mud	88	0.0 [0.0–0.0]	[0.0–1.0]	56	0.9 [0.3–1.0]	[0.0–1.0]	<2.0 10^−16^
Month							
June	80	0.0 [0.0–0.0]	[0.0–0.3]	0	–	–	–
July	80	0.0 [0.0–0.0]	[0.0–0.2]	0	–	–	–
August	40	0.0 [0.0–0.0]	[0.0–0.1]	40	1.0 [1.0–1.0]	[0.9–1.0]	<2.0 10^−16^
September	40	0.0 [0.0–0.0]	[0.0–0.1]	40	1.0 [1.0–1.0]	[0.9–1.0]	<2.0 10^−16^
October	40	0.0 [0.0–0.1]	[0.0–0.2]	40	1.0 [1.0–1.0]	[0.8–1.0]	<2.0 10^−16^
November	40	0.0 [0.0–0.1]	[0.0–0.1]	40	1.0 [0.9–1.0]	[0.1–1.0]	2.6 10^−14^
December	40	0.0 [0.0–0.1]	[0.0–0.1]	40	1.0 [0.9–1.0]	[0.2–1.0]	2.5 10^−15^
January	40	0.0 [0.0–0.1]	[0.0–0.1]	40	0.7 [0.3–0.8]	[0.0–1.0]	1.0 10^−13^
February	40	0.0 [0.0–0.0]	[0.0–0.2]	40	0.3 [0.2–0.5]	[0.0–1.0]	2.5 10^−14^

**Table 2:** Univariate analysis of induced mortality against *Aedes albopictus* in experimental huts

Variables	Untreated huts			Treated huts			*p value*
	N	Median [IQR]	[Min–Max]	N	Median [IQR]	[Min–max]	
		[Q1–Q3]			[Q1–Q3]		
Treatment	440	0.0 [0.0–0.0]	[0.0–0.3]	280	1.0 [0.7–1.0]	[0.0–1.0]	<2.2 10^−16^
Site							
Ambohitranivo	220	0.0 [0.0–0.0]	[0.0–0.3]	140	0.9 [0.7–1.0]	[0.0–1.0]	<2.2 10^−16^
Saharevo	220	0.0 [0.0–0.0]	[0.0–0.3]	140	1.0 [0.7–1.0]	[0.0–1.0]	<2.2 10^−16^
Wall type							
Wood	88	0.0 [0.0–0.0]	[0.0–0.3]	56	1.0 [1.0–1.0]	[0.7–1.0]	<2.2 10^−16^
Cement	88	0.0 [0.0–0.0]	[0.0–0.2]	56	0.8 [0.3–1.0]	[0.0–1.0]	<2.2 10^−16^
Vegetal materials	88	0.0 [0.0–0.0]	[0.0–0.2]	56	1.0 [0.9–1.0]	[0.4–1.0]	<2.2 10^−16^
Tin	88	0.0 [0.0–0.0]	[0.0–0.2]	56	1.0 [0.7–1.0]	[0.0–1.0]	<2.2 10^−16^
Mud	88	0.0 [0.0–0.0]	[0.0–0.3]	56	0.7 [0.2–1.0]	[0.0–1.0]	<2.2 10^−16^
Month							
June	80	0.0 [0.0–0.0]	[0.0–0.1]	0	–	–	–
July	80	0.0 [0.0–0.0]	[0.0–0.2]	0	–	–	–
August	40	0.0 [0.0–0.0]	[0.0–0.2]	40	1.0 [1.0–1.0]	[0.9–1.0]	<2.2 10^−16^
September	40	0.0 [0.0–0.0]	[0.0–0.1]	40	1.0 [1.0–1.0]	[0.9–1.0]	<2.2 10^−16^
October	40	0.0 [0.0–0.1]	[0.0–0.3]	40	0.9 [0.8–1.0]	[0.6–1.0]	4.9 10^−15^
November	40	0.0 [0.0–0.1]	[0.0–0.2]	40	0.9 [0.8–1.0]	[0.5–1.0]	2.5 10^−14^
December	40	0.0 [0.0–0.1]	[0.0–0.1]	40	0.9 [0.6–1.0]	[0.1–1.0]	5.5 10^−12^
January	40	0.0 [0.0–0.1]	[0.0–0.3]	40	0.5 [0.1–1.0]	[0.0–1.0]	3.2 10^−6^
February	40	0.0 [0.0–0.0]	[0.0–0.2]	40	0.4 [0.1–0.7]	[0.0 –1.0]	5.7 10^−9^

The multivariate model showed that the induced mortality of *An. arabiensis*, when compared to wood, is largely reduced when the treatment is sprayed on a porous surface like cement or mud (odds ratio (OR) [95 % confidence interval (CI)]: 0.2 [0.1–0.3] and 0.2 [0.1–0.6], respectively); it is reduced but more modestly when sprayed on tin substrate (OR (95 % CI]: 0.4 [0.2–0.6]). In *Ae. albopictus*, as compared to wood, induced mortality of the insecticide is even more greatly reduced in cement and mud surface (OR [95 % CI]: 0.07 [0.04–0.1] and 0.005 [0.03–0.1], respectively); and more modestly reduced when sprayed on tin substrate (OR [95 % CI]: 0.2 [0.1–0.4]) or on vegetal materials (OR [95 % CI]: 0.5 [0.3–0.8]).

Using the same multivariate model and regardless of the type of pulverized substrate, mortality of *An. arabiensis* decreased significantly from the 4th month after treatment (OR = 1.9 10^−3^ [8.8 10^−5^–4.0 10^−2^]). For *Ae. albopictus*, a significative decrease of mosquito mortality is observed from the 3rd month post treatment (OR = 2.9 10^−3^ [3.1 10^−4^–2.7 10^−2^]) (Table 3).

**Table 3:** Multivariate analysis of induced mortality against susceptible mosquito strains in experimental huts (mixed effect logistic regression)

Species		*An. arabiensis*		*Ae. albopictus*	
Variables	N	Adjusted OR [CI 95 %]	*p value*	Adjusted OR [CI 95 %]	*p value*
Treatment					
No	440	1		1	
Yes	280	1.6 10^5^ [1.1 10^4^–2.6 10^6^]	10^−15^	1.3 10^5^ [1.6 10^4^–1.1 10^6^]	2.2 10^−16^
Wall type					
Wood	144	1		1	
Cement	144	0.2 [0.1–0.3]	10^−6^	0.07 [0.04–0.1]	1.1 10^−12^
Vegetal materials	144	0.6 [0.3–0.9]	0.03	0.5 [0.3–0.8]	0.005
Tin	144	0.4 [0.2–0.6]	10^−3^	0.2 [0.1–0.4]	2.66 10^−6^
Mud	144	0.2 [0.1–0.6]	10^−3^	0.05 [0.03–0.1]	4.4 10^−16^
Month					
August	40	1		1	0.10
September	40	0.9 [0.1–14.2]	0.95	0.2 [0.0–1.4]	0.01
October	40	10.6 [1.5–72.8]	0.02	5.4 [1.5–19.6]	0.06
November	40	32.3 [3.5–294.0]	<10^−2^	3.6 [0.9–13.8]	0.10
December	40	13.4 [1.9–95.3]	0.01	2.6 [0.8–7.8]	<10^−3^
January	40	14.8 [2.2–100.0]	<10^−2^	7.2 [2.3–22.2]	0.43
February	40	7.8 [1.0–61.3]	0.05	1.7 [0.4–7.1]	0.10
Interaction month*insecticide treatment					
August	40	1		1	
September	40	1.0 [0.0–51.5]	0.99	6.0 [0.2–163.0]	0.29
October	40	0.02 [1.1 10^−3^–0.4]	0.01	2.9 10^−3^ [3.1 10^−4^–2.7 10^−2^]	2.7 10^−7^
November	40	1.9 10^−3^ [8.8 10^−5^–0.0]	10^−4^	3.7 10^−3^ [3.7 10^−4^–3.6 10^−2^]	1.5 10^−6^
December	40	1.3 10^−3^ [7.1 10^−5^–0.0]	10^−4^	2.0 10^−3^ [2.3 10^−4^–1.7 10^−3^]	1.3 10^−8^
January	40	2.4 10^−4^ [1.5 10^−5^–4.0 10^−3^]	10^−8^	1.7 10^−4^ [2.0 10^5^–1.5 10^−3^]	6.3 10^−15^
February	40	2.0 10^−4^ [1.1 10^−5^–3.5 10^−3^]	10^−8^	3.7 10^−4^ [3.7 10^−5^–3.7 10^−3^]	1.9 10^−11^

*NS* not significative

**Fig. 4:**
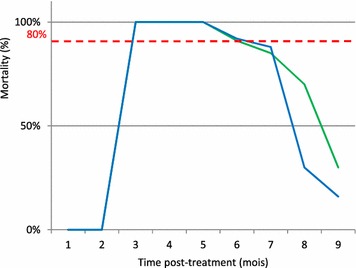
Comparaison of persistence of bendiocarb on mudwalls houses and experimental huts) against *An. arabiensis*

**Fig. 5:**
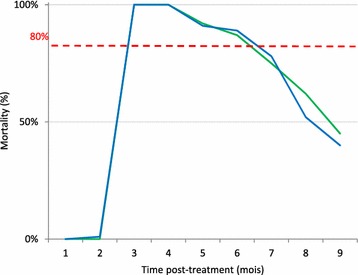
Comparaison of persistence of bendiocarb on mudwalls houses and experimental huts) against *Aedes albopictus*

In houses, for *An. arabiensis* and *Ae. albopictus*, the lethal effect of the insecticide increased significantly compared to control houses without insecticide spraying (p < 0.005) (Tables 4, 5). As expected, mortality rates decreased over time (p < 0.05). For *An. arabien*sis, the mortality rate decreased from 100 to 85 % until the 5th month after treatment and drop from 70 to 30 % at the 6th and 7th months (Fig. 4). For *Ae. albopictus*, mortality rate decrease from 100 to 87 % during 4 months. At the 5th month, the mortality decreased from 75 to 45 % at the 9th month (Fig. 5).

**Table 4:** Univariate analysis of induced mortality against *An. arabiensis* in houses

Variables	Mud households pre-treatment			Mud households post-treatment			*p value* ^a^
	N^b^	Median [IQR]	[Min–max]	N	Median [IQR]	[Min–max]	
Treatment	176	0.0 [0.0–0.0]	[0.0–0.4]	616	0.9 [0.7–1.0]	[0.0–1.0]	<10^−15^
Site							
Ambohitranivo	80	0.0 [0.0–0.0]	[0.0–0.1]	280	0.9 [0.8–1.0]	[0.0–1.0]	<10^−15^
Saharevo	96	0.0 [0.0–0.0]	[0.0–0.4]	336	0.9 [0.7–1.0]	[0.0–1.0]	<10^−15^
Month							
June^c^	88	0.0 [0.0–0.0]	[0.0–0.1]	0	–	–	–
July^c^	88	0.0 [0.0–0.0]	[0.0–0.4]	0	–	–	–
August	0	–	–	88	1.0 [1.0–1.0]	[0.9–1.0]	<10^−15^
September	0	–	–	88	1.0 [1.0–1.0]	[0.9–1.0]	<10^−15^
October	0	–	–	88	1.0 [0.9–1.0]	[0.7–1.0]	<10^−15^
November	0	–	–	88	0.9 [0.9–1.0]	[0.6–1.0]	<10^−15^
December	0	–	–	88	0.9 [0.8–1.0]	[0.6–1.0]	<10^−15^
January	0	–	–	88	0.7 [0.5–0.8]	[0.1–1.0]	<10^−15^
February	0	–	–	88	0.3 [0.2–0.5]	[0.0–0.8]	<10^−15^

^a^Comparaison of the distribution of mortality rate was done with Mann–Whitney-Wilcoxon/Kruskall-Walis test

^b^Effective group

^c^June and July were considered as reference month for pre- and post-treatment data

**Table 5:** Univariate analysis of induced mortality against *Aedes albopictus* in houses

Variables	Mud houses pre-treatment			Mud houses post-treatment			*p value* ^a^
	N^b^	Median [IQR]	[Min–max]	N	Median [IQR]	[Min–max]	
Treatment	176	0.0 [0.0–0.0]	[0.0–0.2]	616	1.0 [0.7–1.0]	[0.0–1.0]	<2.2 10^−16^
Site							
Ambohitranivo	80	0.0 [0.0–0.0]	[0.0–0.0]	280	1.0 [0.7–1.0]	[0.0–1.0]	<2.2 10^−16^
Saharevo	96	0.0 [0.0–0.0]	[0.0–0.2]	336	0.9 [0.7–1.0]	[0.0–1.0]	<2.2 10^−16^
Month							
June^c^	88	0.0 [0.0–0.0]	[0.0–0.1]	0	–	–	–
July^c^	88	0.0 [0.0–0.0]	[0.0–0.2]	0	–	–	–
August	0	–	–	88	1.0 [1.0–1.0]	[0.9–1.0]	<2.2 10^−16^
September	0	–	–	88	0.9 [0.6–1.0]	[0.0–1.0]	<2.2 10^−16^
October	0	–	–	88	0.4 [0.2–0.7]	[0.0–1.0]	<2.2 10^−16^
November	0	–	–	88	0.8 [0.3–1.0]	[0.0–1.0]	<2.2 10^−16^
December	0	–	–	88	0.9 [0.8–1.0]	[0.2–1.0]	<2.2 10^−16^
January	0	–	–	88	1.0 [0.8–1.0]	[0.6–1.0]	<2.2 10^−16^
February	0	–	–	88	1.0 [1.0–1.0]	[0.9–1.0]	<2.2 10^−16^

^a^Comparaison of the distribution of mortality rate was done with Mann–Whitney-Wilcoxon/Kruskall-Walis test

^b^ Effective group

^c^ June and July were considered as reference month for pre- and post-treatment data

In Ambohitranivo and in Saharevo, respectively 93.3 and 86.6 % of house’s wall were made of mud. Comparison of mortality rate in mud experimental huts and mud houses showed no significant difference for the first few months, and then the mortality is significantly higher in houses then in experimental huts (Tables 6, 7). In both conditions, the mortality of *Anopheles* is up to 80 % from the first to the 5th month post-treatment. It is less than 80 % for the 6th and 7th months.

**Table 6:** Comparaison of induced mortality of *An. arabiensis* in mud experimental huts and in mud houses

Month	Experimental huts–mud wall			Houses–mud wall			*p value*
	N	Median [IQR]	[Min–max]	N	Median [IQR]	[Min–max]	
August	8	1.0 [1.0–1.0]	[1.0–1.0]	88	1.0 [1.0–1.0]	[0.9–1.0]	0.61
September	8	1.0 [1.0–1.0]	[1.0–1.0]	88	1.0 [1.0–1.0]	[0.9–1.0]	0.61
October	8	1.0 [1.0–1.0]	[0.9–1.0]	88	1.0 [0.9–1.0]	[0.7–1.0]	0.64
November	8	0.9 [0.9–0.9]	[0.1–1.0]	88	0.9 [0.9–1.0]	[0.6–1.0]	0.84
December	8	0.9 [0.8–1.0]	[0.4–1.0]	88	0.9 [0.8–1.0]	[0.6–1.0]	1.00
January	8	0.3 [0.2–0.4]	[0.2–0.7]	88	0.7 [0.5–0.8]	[0.1–1.0]	0.0007
February	8	0.2 [0.1–0.2]	[0.0–0.3]	88	0.3 [0.2–0.5]	[0.0–0.8]	0.009

**Table 7:** Comparaison of induced mortality of *Aedes albopictus* in mud experimental huts and in mud houses

Month	Experimental huts–mud wall			Houses–mud wall			*p value*
	N	Median [IQR]	[Min–max]	N	Median [IQR]	[Min–max]	
August	8	1.0 [1.0–1.0]	[0.9–1.0]	88	1.0 [1.0–1.0]	[0.9–1.0]	0.69
September	8	1.0 [1.0–1.0]	[0.9–1.0]	88	1.0 [1.0–1.0]	[0.9–1.0]	0.69
October	8	0.8 [0.8–0.8]	[0.6–0.9]	88	1.0 [0.9–1.0]	[0.6–1.0]	<10^−3^
November	8	0.7 [0.6–0.8]	[0.5–1.0]	88	0.9 [0.8–0.9]	[0.2–1.0]	0.006
December	8	0.6 [0.5–0.7]	[0.1–1.0]	88	0.9 [0.6–1.0]	[0.0–1.0]	0.04
January	8	0.1 [0.0–0.2]	[0.0–0.3]	88	0.8 [0.3–1.0]	[0.0–1.0]	<10^−3^
February	8	0.1 [0.0–0.1]	[0.0–0.2]	88	0.5 [0.2–0.7]	[0.0–1.0]	<10^−3^

## Discussion

In the present study, the persistency of insecticide, estimated by observed *An. arabiensis* and *Ae. albopictus* mortality, depended on the type of wall substrate and the time elapsed since the insecticide spraying [17]. Indeed, in addition to enabling the assessment of bio-efficacy and residual activity, the wall bioassays also highlighted how differences in treatment surface substrates can affect insecticidal efficacy. That is to say, efficacy of active ingredients on mosquitoes is modulated by type of substrate onto which the compound is applied [12].

The variation of the residual life of the bendiocarb according to the surface treated observed in the present study also confirms previous observations [11, 12]. As in the present study, Ficam had a good residual activity during five months on both vegetal materials and mud in experimental huts [18]. Bioassay carried out by Ansari et al. [11] in India revealed a persistence of bendiocarb against *Anopheles culicifacies* at 100 % mortality for about 10 weeks on mud whereas Mpofu et al. [18] in Zimbabwe showed that bendiocarb provided 74 % of *An. arabiensis* mortality up to 5 months after spray on mud [11, 18]. In Cameroon, Etang and colleagues reported that 13 weeks after spray on mud, the estimated efficacy of bendiocarb in terms of *Anopheles gambiae s.s.* killing was 80 % [12]. As observed in Mozambique, there were no significant differences in mortality of *An. arabaiensis* evaluated on various porous substrates: mud and cement wall [19]. A similar study in the Philippines [20] found that bendiocarb provided between 75 and 100 % mortality of *Anopheles flavirostris* during the first 3 months post-spray. However, these authors did not specify the surface type onto which the insecticide was sprayed. In Zimbabwe, Mpofu et al. [18] showed that bendiocarb provided a post-spray mortality of 74 % on mud with up to 100 % compared on vegetal materials, 5 months after the initial spray.

Bendiocarb WP showed shorter persistence (3 months) when applied to mud walls. One of the main reasons for the loss of insecticide activity may be the fast absorption by porous surfaces. Mud surfaces are very porous and the application of alkaline substances may degrade the molecule of the insecticide faster [21]. This residual life of bendiocarb is upper than that observed on mud in others studies. For instance, mud surfaces can be highly porous and adsorptive to insecticides, and substrates containing alkaline substances may degrade the candidate insecticide faster than substrates without alkaline contents [12].

A drastic drop in mortality was observed after 4 month in both the Iran and the present trial. From the results of the present study and that in Iran, it appears that the duration of insecticide activity is somewhat more prolonged on vegetal materials than on mud walls. Sprayed on a cement wall, bendiocarb decayed in less than 4 months, showing a short-life but was still considered as a promising insecticide to control resistant vectors as in Benin and in Tanzania [22, 23] Akogbeto et al. [11] suggested that a micro-encapsulation formulation of bendiocarb would make it last longer on treated surfaces. After 4 months experiment of indoor residual spraying treatments in experimental huts in Benin, bendiocarb was shown to be effective in controlling pyrethroid-resistant *Anopheles*, [24]. The useful life of bendiocarb does not exceed 6 months when sprayed on cement-plastered or mud surfaces [19].

Based on present data, as the spray deposits become progressively older, this irritability became less marked and led to mosquito mortalities up to 80 %, so the spraying cycles may not exceed 20 weeks for bendiocarb WP on mud walls while it may last 20 weeks at least for the others types of surfaces.

Based on percentage of mortalities observed in the bioassays, where contact between mosquitoes and sprayed surfaces is ensured, this study shows that activity of bendiocarb can decline significantly within several months after spraying. It doesn’t reflect the purposes of WHO which showed that in many cases IRS of bendiocarb becoming ineffective earlier than the time when they would normally be due for re-spraying.

Chemically induced avoidance behaviors by Malagasy malaria vector mosquitoes should be defined using standardized methods (e.g., excito-repellency boxes and experimental huts) to determine the exact impact of chemicals on malaria transmission and malaria control [25]. Although chemicals used for vector control have historically been evaluated based on toxicity, characterizing the spatial repellent and contact irritant actions of these compounds is a necessity to further the understanding of the mechanism of action of these important public health tools. Such an understanding will help drive innovative methods for disease control using currently available resources as well as aid in the development of novel compounds [26].

The first thoughtfulness to choose the insecticide to be used for IRS is its confirmed effectiveness on the target vector species. It is also crucial to have knowledge of the residual life of insecticide used in IRS program to guess an effectiveness of malaria vector control interventions in Madagascar. Note that IRS in Madagascar is mainly deployed in low-transmission settings and the Deployment of LLIN and IRS may have prevented 100,000 cases annually [27]. Given that the efficacy of bendiocarb decreases below 80 % 5 months after treatment, the spraying cycles of bendiocarb may not exceed 5 months to have a protective effectiveness of IRS using bendiocarb in Madagascar.

In this current study, results showed a similarity obtained during bioassay tests performed in experimental huts and in houses made of muds. Indeed, this is the first study in Madagascar which uses experimental huts as tools for insecticide residual life evaluation. Findings resulting from this study validate that experimental huts tool is a perfect tool to make an extrapolation of insecticide bio-efficacy on walls.

## Conclusion

The bendiocarb is effective against *Anopheles arabiensis* and *Aedes albopictus* sensitive strain tested in the present study. The residual life of this insecticide on walls made of mud in experimental huts and in the village has the same duration. This finding reflects that experimental huts in Madagascar are perfect tool to evaluate the residual life of insecticide used in spraying.

## Abbreviations

DDT: dichlorodiphenyltrichloroethane
IRS: insecticide residual spraying
ITN: insecticide-treated nets
IQR: inter-quartile range
NMCP: National Malaria Control Programme
WHO: World Health Organization
